# Analysis of Differentially Expressed Genes and Signaling Pathways Related to Intramuscular Fat Deposition in Skeletal Muscle of Sex-Linked Dwarf Chickens

**DOI:** 10.1155/2014/724274

**Published:** 2014-03-17

**Authors:** Yaqiong Ye, Shumao Lin, Heping Mu, Xiaohong Tang, Yangdan Ou, Jian Chen, Yongjiang Ma, Yugu Li

**Affiliations:** ^1^College of Veterinary Medicine, South China Agricultural University, Guangzhou, Guangdong 510642, China; ^2^College of Life Science, Foshan University, Foshan, Guangdong 528231, China

## Abstract

Intramuscular fat (IMF) plays an important role in meat quality. However, the molecular mechanisms underlying IMF deposition in skeletal muscle have not been addressed for the sex-linked dwarf (SLD) chicken. In this study, potential candidate genes and signaling pathways related to IMF deposition in chicken leg muscle tissue were characterized using gene expression profiling of both 7-week-old SLD and normal chickens. A total of 173 differentially expressed genes (DEGs) were identified between the two breeds. Subsequently, 6 DEGs related to lipid metabolism or muscle development were verified in each breed based on gene ontology (GO) analysis. In addition, KEGG pathway analysis of DEGs indicated that some of them (GHR, SOCS3, and IGF2BP3) participate in adipocytokine and insulin signaling pathways. To investigate the role of the above signaling pathways in IMF deposition, the gene expression of pathway factors and other downstream genes were measured by using qRT-PCR and Western blot analyses. Collectively, the results identified potential candidate genes related to IMF deposition and suggested that IMF deposition in skeletal muscle of SLD chicken is regulated partially by pathways of adipocytokine and insulin and other downstream signaling pathways (TGF-**β**/SMAD3 and Wnt/catenin-**β** pathway).

## 1. Introduction

In the past decades, poultry breeding predominantly focused on increasing growth rate and meat yield and improving body composition by producers. Poultry production has dramatically increased, meeting both consumer demand and commercial profit requirements desired by producers; however, the impressive progress made in these traits has been accompanied by deterioration of taste quality of the meat which leads consumers to seek better tasting chicken meat [1–3]. For instance, the SLD chicken, which has a 1773-bp deletion mutation in the 3′UTR of Growth Hormone Receptor (GHR), possesses excellent meat taste and has been accepted by many people, but it has an inferior growth rate, decreased body weight, and increased fat content of muscle compared with the normal chicken [4, 5].

IMF is mainly distributed in the epimysium, perimysium, and endomysium and accumulated between muscle fibers or within muscle cells. Previous studies demonstrated that a certain amount of IMF can enhance meat quality, such as the flavor, juiciness, water holding capacity, and tenderness [6–8]. Thus, IMF is an important evaluation index for the meat quality of chicken. Plenty of studies revealed that the differentiation of preadipocytes into adipocytes induces gene expressions of two master transcription factors, CCAAT/enhancer binding protein (C/EBP) and peroxisome proliferator-activated receptor (PPAR) using 3T3-L1 preadipocytes [9–12]. However, no study has identified the key regulator related to adipogenesis within the skeletal muscle of chicken.

For the molecular mechanism of IMF deposition, numerous studies have been performed on livestock [13–17], Beijing-you (BGY), and Arbor Acres (AA) chickens [18, 19], but studies on SLD chicken have not been reported. Therefore, the discovery and characterization of genes that are differentially expressed between the SLD and normal chicken would be a useful tool for identification of the IMF regulators in muscle of chicken.

In the present study, characterization of mRNA expression profiles in skeletal muscles of 7-week SLD and normal chickens was performed using Affymetrix chicken gene chips and 6 candidate genes that may affect IMF deposition were selected out. Then, the expression changes of 6 candidate genes and other adipogenesis-related signaling pathway genes were measured by using qRT-PCR and/or Western blot analysis to examine the association of those genes with IMF deposition in skeletal muscle of SLD chicken.

## 2. Material and Methods

### 2.1. Animals

SLD and normal recessive White Rock chickens, both bred for nearly 10 generations, were used. Dwarf chickens had a 1773-bp deletion mutation at the end of exon 10 and in the 3′UTR of GHR. The weight of dwarf chickens was about 30% less than that of normal chickens. The two strains were fed under the same conditions (ad libitum feeding, the same food stuff) to 7 weeks of age. All animal experiments involved in this study were approved by the Animal Care Committee of South China Agricultural University (Guangzhou, China). Chickens were euthanized as necessary to ameliorate suffering.

### 2.2. Sample Collection

Nine birds of similar weight from each breed were sacrificed for tissue collection. Samples of the left leg gastrocnemius muscles were excised, divided into three parts, placed into cryopreservation tubes, and quickly snap-frozen in liquid nitrogen (−196°C) for preservation. The entire right leg muscle was collected and stored at −20°C for IMF content measurements.

### 2.3. Measurement of Lipid Contents in Leg Muscle of Dwarf and Normal Chicken

IMF content of leg muscle was determined by the Soxhlet method according to previous studies [20], using anhydrous ether as the solvent, and expressed as percentages of the dry weight.

### 2.4. Extraction of Total RNA

Total RNA was isolated from skeletal muscle tissues with TRIzol (Takara Biotech Co. Ltd., Dalian, China) according to the manufacturer's instructions. The purity and yield of RNA were determined using optical density at 260 and 280 nm. RNA integrity was examined by electrophoresis on a 1.2% denaturing formaldehyde gel.

### 2.5. Microarray Analysis

Three pools of RNA were prepared for each chicken strain, with each pool containing RNA from three individuals. Microarray hybridization was carried out by Affymetrix Inc. (Beijing, China) using Agilent chicken gene chips with 38535 probes. The DEGs were selected out by using Significance Analysis of Microarrays (SAM) software, and the screening criteria were as follows: *q*-value ≤0.05; with a fold change ≥2; or a fold change ≤0.5. Then the gene ontology enrichment analysis was performed for function corresponding to DEGs in chicken using the GOEAST software toolkit (*P* ≤ 0.05), signaling pathway analysis was carried out using KEGG data software, and the genes related to adipogenesis were selected. Finally, the enrichment analyses of DEGs were performed by using the DAVID 6.7 software.

### 2.6. qRT-PCR Analysis

To validate the microarray hybridization results, 6 genes were selected from the DEG list for qRT-PCR assays. In addition, 16 adipogenesis-related signaling pathway genes were analysed in the RNA samples by qRT-PCR. Using published genome sequences, the Primer Premier 5 software was used for primer design (Supplemental file 1 in the Supplementary Material available online at http://dx.doi.org/10.1155/2014/724274). In the present study, the Ct value was applied to detect the mRNA expression of the samples, and three replicates were set for each sample. The thermal cycling protocol was as follows: 95°C for 1 min, then 40 cycles of 95°C for 15 s, appropriate annealing temperature for 45 s, and 72°C for 45 s. The final step after cycling was an extension at 72°C for 40 s. Melting curve analysis was carried out to determine the specificity of PCR products. The 2^−ΔΔCT^ method was used to measure gene expression with *β*-actin as the reference gene.

### 2.7. Western Blot Analysis

The Western blot analysis was performed as described previously [21]. Briefly, the leg muscle was lysed in RIPA buffer supplemented with protease and phosphatase inhibitor mixture (Sigma, USA), and protein concentrations of cell lysates were determined by BCA kit (Beyotime, Haimen, China). The lysates were diluted with sample buffer, separated on 4–20% Tris-HCl/SDS-polyacrylamide gels, and transferred to polyvinylidene fluoride membranes (PVDF; Millipore, USA). The blots were then incubated with mouse monoclonal antibody anti-TGF-*β*3 (Santa Cruz, USA), rabbit polyclonal antibody anti-*β*-catenin (Invitrogen, USA), rabbit polyclonal antibody anti-SMAD3 (Abcam, USA), mouse monoclonal antibody anti-PPAR-*α* (Abcam, USA), and rabbit polyclonal antibody anti-GAPDH (Santa Cruz, USA). Immune complexes were visualized by incubation with specific secondary antibodies conjugated to HRP (horseradish peroxidase; Santa Cruz, USA) and membranes were detected with BeyoECL Plus kit (Beyotime, Haimen, China). Imaging was performed with Bio-Rad imaging system (Bio-Rad, USA), and the band intensities were analysed via the Image J software (Bio-Rad, USA). The mean intensities of the bands from samples and interference were calculated. The relative expression of the target protein was valuated with the gray value ratio of target protein content to GAPDH (target protein/GAPDH) content.

### 2.8. Statistical Analysis

Results are presented as mean ± SEM, and qRT-PCR experiments included at least eight biological replicates per group and three technological replicates. Data were evaluated using a two-tailed Student's *t*-test, and differences between groups were considered statistically at *P* < 0.05. All statistical analyses were performed with SPSS 17.0 software.

## 3. Results and Discussions

### 3.1. Body Weight and IMF Content between the Two Chicken Lines

**Figure 1:**
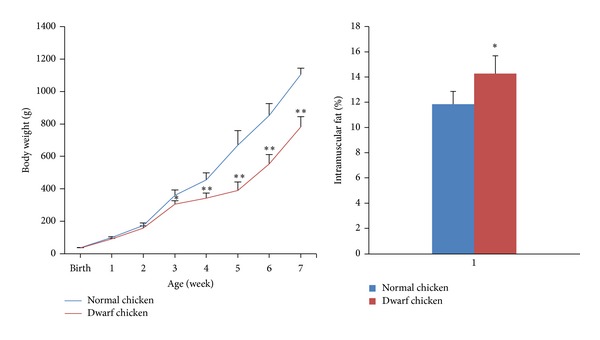
Body weight and IMF percentages of the dwarf and normal chickens. Significant differences in body weight between the two lines were apparent from the 3rd week; the IMF contents in dwarf chickens are significantly higher than those in normal chickens. Note: *P < 0.05, **P < 0.01 versus normal groups (*n* = 30).

IMF content in the dwarf chickens is 1.2 times than that in the normal chickens (Figure 1). Our results are consistent with previous studies that deletion mutation in 3′UTR of GHR can result in a significant reduction in body weight and increased adiposity and IMF contents, indicating that these two chicken models provide a good model to study IMF deposition.

### 3.2. Microarray Data Analysis

Recessive sex-linkage dwarf gene (dw) is the only recessive mutant gene known to be benefit to human and has no harm to chicken health. SLD is perhaps the best characterized model of dw phenotype. Studies indicated that the SLD phenotype is caused by a mutation in the GHR gene that can result in a significant reduction in body weight, insulin-like growth factor 1(IGF1) levels, and increased adiposity and IMF content [4, 5]. The latter, IMF, is responsible for sensory aspects of high meat quality in dwarf chickens. Although profiles of gene expression have been reported on liver, visceral tissues, or muscle cells of chicken [22–24], studies on IMF deposition in dwarf chicken have not been reported. Our study is the first to explore gene expression profiles in skeletal muscle tissues using both dwarf and normal breeds. The present objective was to identify candidate genes and potential pathways that may have some relevance to IMF deposition in dwarf chicken.

In our study, a total of 38,535 probes were used to detect mRNA expression profiles in chicken skeletal muscles, of those the probes displaying hybridization signals represented approximately 42.62–45.6% of the total; approximately 52.8–55.7% of probes lacked hybridization signals and about 1.5–1.7% of probes showed ambiguous hybridization signals (Table 1).

**Figure 2:**
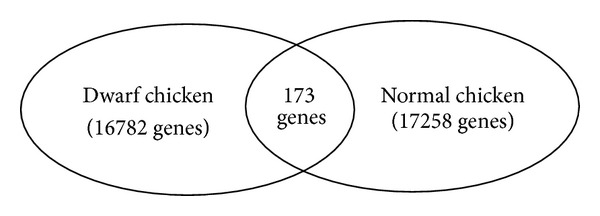
Numbers of genes that were differentially expressed in skeletal muscles between dwarf and normal chickens.

From our results, 16782 and 17285 genes were detected as expressed genes in dwarf and normal chickens, respectively (Figure 2). Of those, 173 DEGs (57 known) were shared by the two breeds, where 65 genes were upregulated and 108 genes were downregulated in dwarf chickens compared with the normal chickens (Supplemental file 2). The gene expressions of GHR, SLC25A30, OSGIN1, and NPTX2 in dwarf chickens were upregulated 5-fold or more than 5-fold, and the expressions of HSPA8, CRISPLD2, and AANAT were upregulated 4-fold or more than 4-fold than in normal chickens, while the fold changes of the following genes in dwarf chickens were significantly downregulated compared with those in normal ones: SUCLG2 (25-fold), LOC770114 (16-fold), RCJMB04_1f9 (12-fold), ACY1L2 (10-fold), LOC776458 and OTOR (5-fold), ENPP4, CA5B, and RCJMB04_35 g11/VNN1 (4-fold), indicating that those genes may play key roles in skeletal muscle development of dwarf chickens.

Based on the known DEGs, GO analyses were performed in each breed, and the enriched GO-terms (*P* < 0.05) analysis in the ontology classification “biological process” was selected and is presented in Supplemental file 3. The results showed that the biological process that was shared by the two breeds mainly included the following process: immune system development, skeletal muscle growth, hormone metabolism, protein metabolism, lipid metabolism, regulation of cell differentiation and apoptosis, transcription factor activity, regulation of hemopoiesis-related hemopoietic and blood circulation, and regulation of RNA metabolic process, extracellular region, and heparin binding.

Through the gene enrichment analysis, BCL6 was found be to the most frequent gene involved in those biological functions, then were JMJD6 and KIT, and followed by CBFB, MB, HOXA3, PTN, GHR, ARNT, HLF, ICER, LOC417056, LOC417083, YFVI, MR1, AGTR1, CPZ, FGF1, FOXK2, LOC396260, NR1D2, OTOR, PON2, POSTN, MAFF, RORA, ST6GAL1, and TGIF1 genes (Supplemental file 4), suggesting that those genes may participate in the regulation of chicken skeletal muscle development with high frequency.

### 3.3. Key Genes Related to Lipid Metabolism or Muscle Development

According to Cui et al. [19], in skeletal muscle, genes that are related to lipid metabolism or muscle development would contribute to IMF deposition. The GO-term analysis showed that 6 known DEGs related to lipid metabolism or muscle development were differentially expressed between the two breeds including insulin-like growth factor binding protein 3 (IGF2BP3), thyroid hormone-responsive protein, Spot14 homology (THRSP), nuclear receptor subfamily 1, group D, member 2 (NRID2, also known as Rev-erb*β*), RAR-related orphan receptor A (ROR*α*), suppressor of cytokine signaling 3 (SOCS3), and GHR.

IGF2BP3, one of the important members of insulin-like growth factor RNA binding protein family (RNA-binding proteins, RBPs), has pro-growth functions by binding to IGFs [5]. The GHR is critical receptor for Growth Hormone (GH), which function in promoting body development and fat deposition by activating intracellular or intercellular signal transduction pathway via combining with GHR [25]. SOCS3, a member of SOCS family, is a key determinant of basal insulin signaling and is an important molecular mediator of cytokine-induced insulin resistance in adipocytes [26]. ROR*α* and NR1D2 are critical regulators of circadian rhythm clock with significant roles in lipid homeostasis. ROR*α* activates brain muscle arnt-like protein-1 factor (Bmal1) transcription and mediates lipogenesis and lipid storage in skeletal muscle [27–29]. NR1D2 can repress Bmal1 transcription and is involved in adipocyte differentiation not only in adipose tissue but also in skeletal muscle, liver, and brain [30, 31]. THRSP, one of the genes that were mediated by thyroid hormone in nucleus, has close relationship with IMF content [14, 32].

### 3.4. qRT-PCR and Western Blot Analysis

To further validate the results of microarray testing, qRT-PCR was used to examine the relative expression of 6 DEGs selected in each breed. As shown in Table 2, fold changes in gene expression between the two methods were correlated in both dwarf and normal chickens.

Next, the mRNA or protein levels of other downstream genes that related to adipogenesis (Supplemental file 1) were also measured by using qRT-PCR and Western blot analysis.

**Figure 3:**
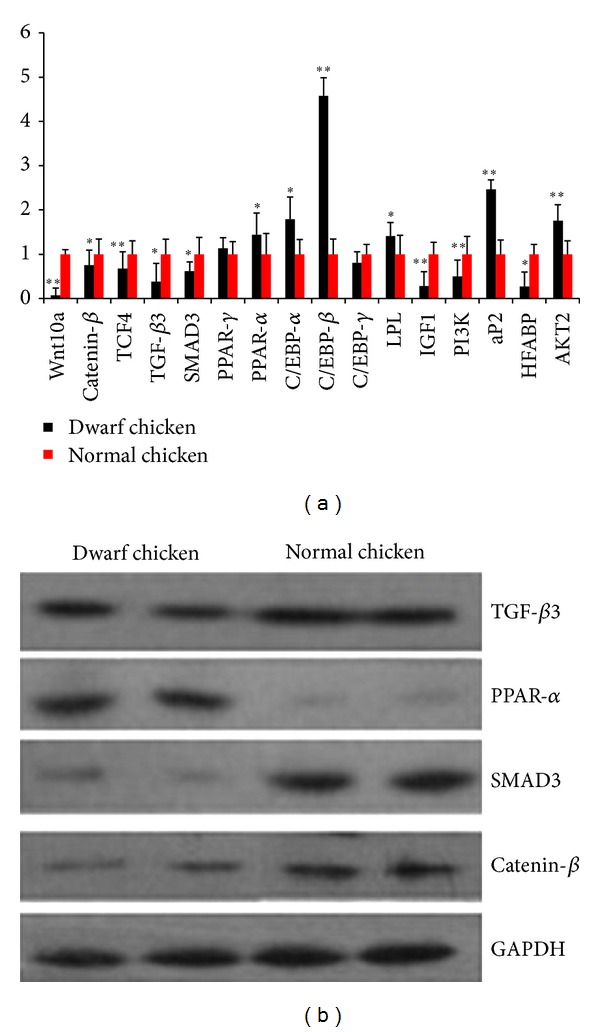
(a) The mRNA expression of some signaling pathway factors was measured by qRT-PCR. Data were normalized using *β*-actin mRNA and were presented as the mean ± S.E.M of the three experiments, *n* = 10/group. Data are representative of three separate experiments. Note: *P < 0.05, **P < 0.01 versus normal groups. (b) Western blot analysis of indicated proteins: catenin-*β*, TGF-*β*3, SMAD3, and PPAR-*α* protein expression in normal and dwarf chicken skeletal muscles. GAPDH was used as loading and transfer control. Representative blots are shown.

As shown from Figure 3(a), the mRNA expressions for some proadipogenesis factors *α*P2, PPAR-*α*, PPAR-*γ*, C/EBP-*α*, C/EBP-*β*, and LPL in dwarf chickens were upregulated while some antiadipogenesis signaling factors Wnt10a, TCF4, catenin-*β*, SMAD3, TGF-*β*3, C/EBP-*γ*, and HFABP mRNA were downregulated compared with normal chickens. In addition, the expressions of insulin signaling pathway genes IGF1 and PI3 K were also downregulated, and the AKT2 was upregulated.  Figure 3(b) showed the levels of protein expression of catenin-*β*, SMAD3, TGF-*β*3, and PPAR-*α* in skeletal muscle between dwarf and normal chickens and the results are consistent with the mRNA expression, indicating that mRNA levels adequately represent protein levels.

### 3.5. Potential Pathways Related to IMF Deposition

KEGG pathway analysis was performed on those 6 DEGs to explore the potential pathways that may relate to IMF deposition in dwarf and normal chicken. The results showed that IGF2BP3 is associated with the insulin signaling pathway; both SOCS3 and GHR are associated with adipocytokine and insulin signaling pathways and SOCS3 is also involved in the regulation network of GHR gene.

**Figure 4:**
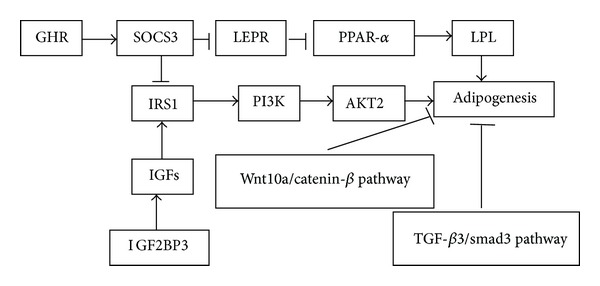
Schematic illustrations for potential signaling pathway of IMF deposition in skeletal muscles regulated by GHR and SOCS3. GHR increases the expression of SOCS3. SOCS3 affected adipogenesis by inhibiting the expression of LERP and IRS1, participating in adipocytokine signaling pathway. IGF2BP3 affected insulin signaling pathway by regulating IGFs.

In adipocytokine signaling pathway, SOCS3 affects adipogenesis perhaps by regulating two downstream genes, lipophorin receptor (LEPR) and insulin receptor substrate 1(IRS1). On the one hand, SOCS3 increases PPAR-*α* expression by inhibiting LEPR, affecting the function of leptin and fatty acid metabolism. On the other hand, SOCS3 inhibits the phosphorylation of IRS1 affecting insulin signaling (Figure 4).

qRT-PCR analysis in our previous study [33] has shown that mRNA expression of GHR and SOCS3 in the adipocytokine pathway was increased and IGF1 and IGF2BP3 in the insulin pathway were decreased, respectively, in dwarf chickens compared with normal chickens. Meanwhile, expression of IRS1 and LEPR, downstream genes of adipocytokine signaling pathway, was also downregulated [33]. The qRT-PCR and Western blot results in our study showed that adipogenesis-related factors (C/EBP-*α*, C/EBP-*β*, *α*P2, LPL, and THRSP), which have been demonstrated to have relevance for in vivo adipogenesis [34–38], were upregulated significantly. Moreover, both the mRNA and protein levels of PPAR-*α*, downstream gene of LEPR, were also increased, suggesting that adipocytokine signaling pathway could play prominent role in IMF deposition of dwarf chicken.

In insulin signaling pathway, for dwarf chicken, upregulated SOCS3 inhibits the phosphorylation of IRS1 affecting insulin signaling. Previous studies have demonstrated that Wnt10a/catenin-*β* pathway can repress IMF deposition [39, 40] and that TGF-*β*3/Smad3 signaling also plays a critical role in inhibiting adipogenesis and can interact with Wnt/catenin-*β* pathway [37, 41], probably playing a role in the downstream of insulin signaling. In addition, IGF2BP3 affected insulin signaling pathway by combining with IGFs. In our result, the expressions of Wnt10a, catenin-*β*, TCF4, TGF-*β*3, SMAD3, and PI3 K mRNA were all downregulated in dwarf chicken compared with normal chicken, but the expression of AKT2 mRNA was upregulated in insulin pathway, probably due to the fact that AKT2 was also regulated by other genes and pathways. Moreover, the protein levels of catenin-*β*, TGF-*β*3, and SMAD3 were also decreased sharply, and this may be helpful in supporting the significant role of adipocytokine signaling and insulin signaling in IMF deposition of chicken.

### 3.6. ROR*α* and NR1D2 May Affect IMF Deposition

ROR*α* and NR1D2 have been implicated in affecting lipid metabolism [27–30]. Loss-of-function studies by Ramakrishnan et al. have identified some proadipogenesis factors such as ap2/CD36 and PPAR-*α* as target of NRID2 [27]. In our study, the mRNA of ROR*α* and NR1D2 was significantly downregulated and upregulated in skeletal muscles of dwarf chickens, respectively. The increased NR1D2 expression further induces expression of other downstream adipogenesis-related candidate genes, PPARs, C/EBP-*α*, aP2, and LPL, and finally affects IMF deposition.

The present approach has used gene expression profiling to analyse the DEGs and used qRT-PCR and Western blot analyses to elucidate the molecular events of IMF deposition in chickens. Possibly regulated by multiply signaling pathways and modifications of circadian rhythms-related genes may also contribute.

## 4. Conclusion

In the present study, gene expression profiles of skeletal muscle sampled at 7 weeks old from dwarf and normal chickens were characterized and 173 DEGs were selected out between the two groups. Six DEGs, whose expressions were verified by qRT-PCR and Western blot analysis in both two groups, were tentatively revealed to play key roles in developmental processes of IMF since they participate in adipogenesis-associated signaling pathway. Therefore, the IMF deposition in chickens was proposed to be partially regulated by multiple signaling pathways and circadian rhythms-related genes. The findings obtained in the current study could provide meaningful information for the establishment of the groundwork to further explain the molecular mechanisms underlying IMF deposition in chicken.

## Supplementary Material

Supplemental file 1. Sequences of primers used for qRT-PCR.
Supplemental file 2. The mRNA differential profiles in skeletal muscles of 7-week-old dwarf and normal chickens.
Supplemental file 3. Common Enriched GO terms among the differentially expressed genes in both dwarf and normal chickens.
Supplemental file 4. The frequency of DEGs involved in biological process.

## Figures and Tables

**Table 1:** Summary of gene expression in skeletal muscles of dwarf and normal chickens determined by microarray analysis.

Hybridization signals	Normal chickens			Dwarf chickens		
	A1	A2	A3	B1	B2	B3
Present						
Probes	17174	17124	17559	16506	17417	16425
%	44.6	44.4	45.6	42.8	45.2	42.6
Absent						
Probes	20764	20839	20335	21428	20513	21448
%	53.9	54.1	52.8	55.6	53.2	55.7
Marginal						
Probes	597	572	641	601	605	662
%	1.5	1.5	1.7	1.6	1.6	1.7

Total probes	38535	38535	38535	38535	38535	38535

**Table 2:** Comparison of microarray and qRT-PCR fold changes for selected genes in skeletal muscles of dwarf and normal chickens.

Gene	Fold change (microarray) dwarf/normal	Fold change (qRT-PCR) dwarf/normal	Tendency
GHR	5.263	1.727	Consistency
SOCS3	2.439	2.428	Consistency
THRSP	2.273	2.580	Consistency
ROR*α*	0.433	0.533	Consistency
IGF2BP3	0.33	0.400	Consistency
NR1D2	2.381	6.525	Consistency
